# Complete Genome Sequence of *Rhynchophorus ferrugineus* Endocytobiont “*Candidatus* Nardonella dryophthoridicola” Strain NardRF

**DOI:** 10.1128/MRA.00355-21

**Published:** 2021-07-01

**Authors:** Bessem Chouaia, Matteo Montagna, Pompeo Suma, Franco Faoro

**Affiliations:** a Department of Molecular Sciences and Nanosystems, Ca' Foscari University of Venice, Venice, Italy; b Department of Agricultural and Environmental Sciences, University of Milan, Milan, Italy; c Interuniversity Center for Studies on Bioinspired Agro-Environmental Technology (BAT Center), Università di Napoli Federico II, Portici, Italy; d Department of Agriculture, Food and Environment, University of Catania, Catania, Italy; Indiana University, Bloomington

## Abstract

We report the complete genome sequence and annotation of “*Candidatus* Nardonella dryophthoridicola” strain NardRF, obtained by sequencing its host bacteriome, Rhynchophorus ferrugineus, using Oxford Nanopore technology.

## ANNOUNCEMENT

**FIG 1:**
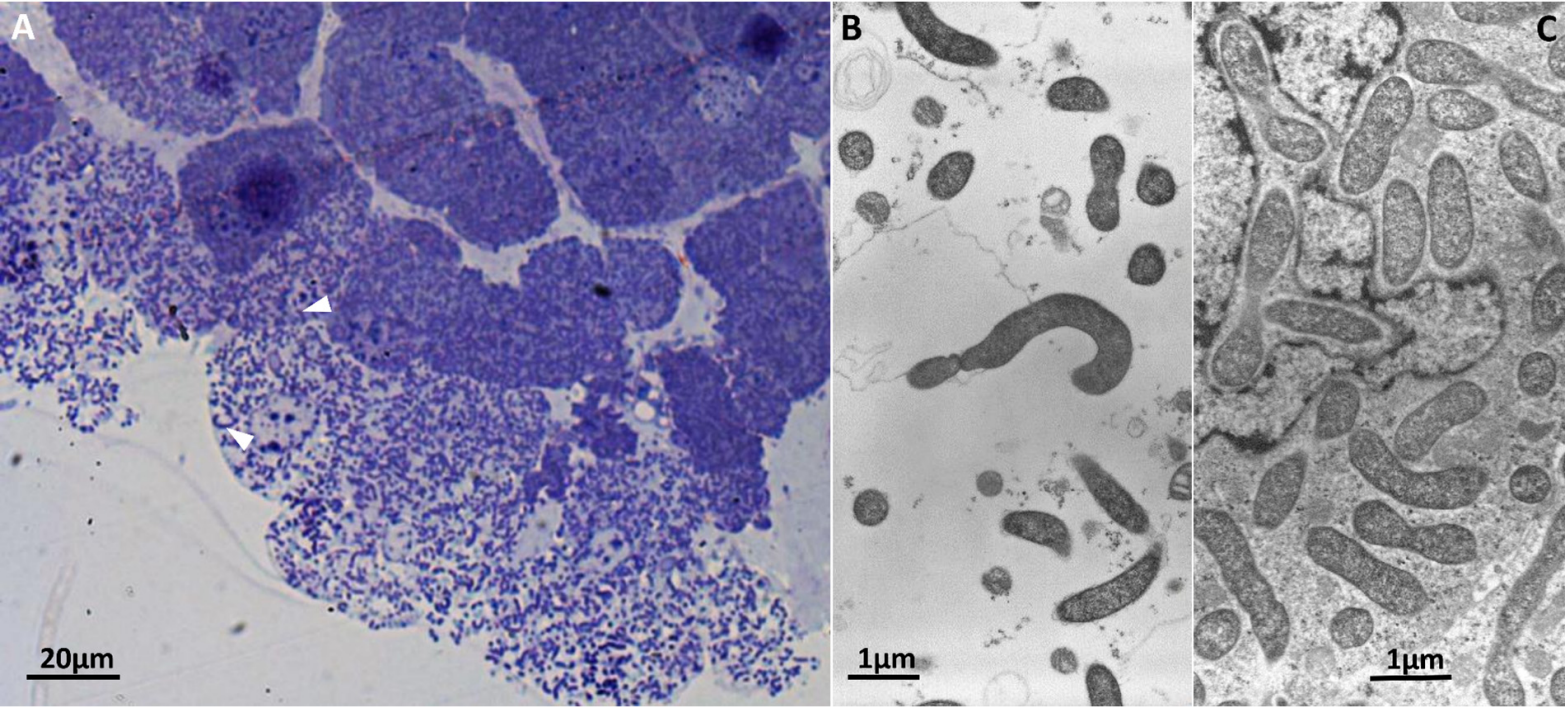
The endocytobiont “*Candidatus* Nardonella dryophthoridicola.” (A) Semithick cross-section of the *Rhynchophorus ferrugineus* bacteriome in which it is possible to observe the bacterial cells, stained with toluidine blue, within the host cell (white arrowheads). (B and C) Ultrathin sections of the same bacteriome under transmission electron microscopy (TEM) showing “*Ca.* Nardonella” rod-shaped cells outside (B) and within (C) the host cell.

The bacterium “*Candidatus* Nardonella dryophthoridicola” is a Gram-negative gammaproteobacterial endocytobiont (Fig. 1). Specifically, it is an intracellular obligate mutualist associated with weevils (1). The bacterium plays a crucial role in cuticle hardening by supplying tyrosine to its host (2). Unlike the second weevil-associated symbiont, “*Candidatus* Sodalis pierantonius,” it is maintained within a functional bacteriome for its host’s entire life cycle (3–5).

We used long-read sequencing to investigate the genome sequence of “*Ca.* Nardonella dryophthoridicola” strain NardRF, associated with an Italian population of Rhynchophorus ferrugineus. The insect hosts were sampled from a single palm tree in the region of Catania in 2017. The pupae were kept at 25°C, 24-h dark, until molting into adults. Ten newly emerged adults were dissected to extract their bacteriomes. The bacteriomes were then pooled for DNA extraction using the DNeasy blood and tissue kit (Qiagen, Italy) following the manufacturer’s instructions for animal tissue extraction. The DNA integrity was verified by 0.8% agarose gel electrophoresis at 90 V for 1 h. The DNA purity and concentration were measured with a NanoDrop 100 spectrophotometer (Thermo Fisher Scientific, Italy) and Qubit double-stranded DNA (dsDNA) high-sensitivity assay kit.

Long-read sequencing was performed using the R9.5 flow cell on a MinION Mk1B device. For the library preparation, 2.5 μg of nonsheared and non-size-selected total genomic DNA was used following the 1D ligation sequencing kit (SQK-LSK 108) protocol. Then, 0.5 μg of the final DNA was loaded onto the flow cell. The sequencing was run for 48 h using MinKNOW v18.03.1. Base calling was then run on the fast5 files using Guppy v4.4.1 (6) with the high-accuracy algorithm and a quality cutoff of 7. Reads longer than 500 bp were used for the subsequent analyses. All tools were run with default parameters unless otherwise specified.

The metagenomics fastq reads (host and symbiont) were first assembled using miniasm (7). Contigs identified as “*Ca.* Nardonella dryophthoridicola” were identified using BLASTn (E value cutoff, 10^−6^) against the NCBI nonredundant (nr) database. These contigs were extracted and used to refine the assembly. The contigs were used to map and extract the “*Ca.* Nardonella dryophthoridicola” long reads using minimap2 v2.17 (8). The 836,116 reads were then reassembled using Flye v2.8.1 (9). The resulting genome was circularized using Circlator v1.5.5 (10) with the options –merge_min_id 85 and –merge_breaklen 1000 as advised for Oxford Nanopore reads. The circular genome was corrected using the publicly available Illumina short reads (SRA accession number SRR12633329 [11]) with POLCA (MaSuRCA v4.0.1) (12, 13). During the different assembly, circularization, and polishing steps, the genome quality was assessed using BUSCO v4.1.4 (14) with the Gammaproteobacteria database. The final genome was automatically annotated using GenBank with PGAP r2021-01-09.build5126 (Table 1) (15).

**TABLE 1:** “*Candidatus* Nardonella dryophthoridicola” strain NardRF long-read and genomic summary features

Feature	Data for:	
	Metagenome	Strain NardRF
Long-read features		
No. of reads	3,474,690	836,116
Mean read length (bp)	2,021	2,018
Longest read (bp)	114,533	88,252
Shortest read (bp)	500	500
*N*_50_ (bp)	3,035	2,991
Genome features		
Size (bp)	NA^*a*^	200,313
GC content (%)	NA	15.33
No. of genes	NA	231
No. of CDS^*b*^	NA	199
No. of RNAs	NA	32
No. of ribosomal operons	NA	1

a NA, not applicable.

b CDS, coding DNA sequences.

Genome comparison with the closest genome (RefSeq accession number NZ_AP018161 [2]), using ACT (Artemis v18.1.0 [16]), revealed that the gene encoding the isoleucine tRNA ligase (*ileS*) was complete in our genome, while containing a 1-nucleotide frameshift at position 820. This difference demonstrates the importance of sequencing the same streamlined bacterial endocytobiont from different host populations, as genome reduction through random genetic mutations combined with a maternal transmission bottleneck can result in genomic differences within the same endosymbiont species.

### Data availability.

The assembly has been deposited in GenBank under accession number CP069383 and BioProject accession number PRJNA699994. The version described in this paper is the first version, CP069383.1. The Oxford Nanopore reads used for the assembly of “*Ca.* Nardonella dryophthoridicola” have been deposited under SRA accession number SRR14598013.
